# Developing a competency model for telerehabilitation therapists and patients: Results of a cross-sectional online survey

**DOI:** 10.1371/journal.pdig.0000710

**Published:** 2025-01-03

**Authors:** Anna Lea Stark-Blomeier, Stephan Krayter, Christoph Dockweiler

**Affiliations:** 1 Department Digital Health Sciences and Biomedicine, School of Life Sciences, University of Siegen, Siegen, Germany; 2 Chair of Information Systems, esp. IT for the Aging Society, School of Economic Disciplines, University of Siegen, Siegen, Germany; Iran University of Medical Sciences, ISLAMIC REPUBLIC OF IRAN

## Abstract

Telerehabilitation is a new form of care that provides digital access to rehabilitative services. However, it places many demands on the users–both patients and therapists. The aim of this study was to determine the requirements and competencies needed for successful usage, identify person- and context-specific differences and develop a competency model. We conducted two cross-sectional online surveys with telerehabilitation patients and therapists from Germany during June-August 2023. The adjusted dataset of 262 patients and 73 therapists was quantitatively analyzed including descriptive and bivariate statistics. Group differences were assessed using t-tests or U-tests. The development of two telerehabilitation competency models was guided by a competency modeling process. The surveys show that patients need to gather program information before program start, follow therapist’s instructions, adapt therapy, deal with health problems, as well as motivate and remind oneself during the program. Therapists need to inform and instruct patients, adapt therapy, carry out technical set-up and support, give medical support, guide and monitor patients, give feedback, motivation and reminder, as well as documentation. The competency model for patients includes 23 and the model for therapists 24 core competencies, including various required areas of knowledge, skills, attitudes and experiences. The three most relevant competencies for patients are self-interest in the program, self-awareness and self-management. Also, disease severity, age, and language abilities can enable successful execution. Program type, technology affinity, and age significantly influence the rated relevance of competencies. The three most relevant competencies for therapists are therapeutic-professional skills, medical and telerehabilitation knowledge. The type of therapy practiced and language abilities can enable successful execution. Therapist’s age, technology affinity, and job type significantly impact the rated relevance. The models should be applied to develop tailored training formats and support decisions on the selection of suitable therapists and patients for telerehabilitation.

## Author summary

When therapists and patients use digital tools to carry out rehabilitation remotely, this is known as telerehabilitation. It can include apps with movement exercises or video conferencing for psychotherapy. In an online survey, we asked therapists and patients from Germany what steps telerehabilitation involves, what competencies are essential and how this differs for different people or circumstances. We analyzed answers of 262 patients and 73 therapists. We found that therapists and patients take different steps to prepare for and perform the therapy (e.g. give/find motivation for remote therapy). For a successful digital therapy, patients must be interested in the program and be able to manage themselves and their behavior. For different programs different competencies are important. Therapists must have good professional skills and knowledge of medicine and telerehabilitation. How important skills are considered differs depending on the age of therapists. Our study is important because patients and therapists can now receive suitable trainings or decide whether telerehabilitation is the right therapy.

## Introduction

Rehabilitation is proclaimed as a key health strategy of the 21st century [1] that “leads to better health, participation, and quality of life of persons with chronic disease or impairment” [2]. Internationally, the design and implementation of rehabilitative systems varies. A basic distinction can be made between medical and vocational rehabilitation. While medical rehabilitation aims to restore the functional and mental abilities and quality of life of people with an illness or disability, vocational rehabilitation focuses on enabling ill or disabled people to overcome barriers to access, remain in or return to work [3,4]. One possibility for securing therapeutic successes achieved in rehabilitation and supporting the transfer of what has been learned into everyday life is rehabilitation aftercare, which is a service that is specifically provided in Germany [5] but is not a standard service in many other countries [6].

Globally, the need for rehabilitation services is largely unmet, for example there are gaps in care in rural areas and overall there are long waiting times [7]. Telerehabilitation as a domain of telehealth presents an opportunity to provide alternative access to rehabilitation services via information and communication technologies [8–10]. Technologies can be used synchronously, e.g. in the form of video conferencing between therapists and patients, or asynchronously, e.g. in the form of time-independent smartphone use [11]. Innovative methods such as virtual reality, tele-robotics and video gaming are also already being used [12]. Telerehabilitation is already being provided in various therapeutic disciplines such as physiotherapy, speech therapy or occupational therapy [12]. In Germany, digital services have been used in particular in the area of rehabilitation aftercare [13]. According to the current state of research, telerehabilitation is comparable to face-to-face rehabilitation regarding its effectiveness, e.g. in the area of in physical therapy [10,14] or cardiac rehabilitation [15].

Despite evidence of the effectiveness of telerehabilitation, there are some barriers to its use and implementation. For example, the lack of user skills or digital literacies are seen as barriers [12]. In a recent scoping review, we found that telerehabilitation requires a variety of different competencies from patients and therapists–going beyond the sphere of technical skills [16]. These competencies comprise various knowledge areas, skills, experiences, attitudes and other personal characteristics defined as enabling behaviors that influence effective performance [16–18]. More research need is stated regarding the specific competencies required for telerehabilitation [16]. This is where competency models, defined as “an organizing framework that lists the competencies required for effective performance in a specific job, […] organization, function, or process” [17], are of great value. While various competency models for healthcare providers such as nurses or physicians have already been developed for the broader discipline of telehealth, this is still outstanding for the use of telerehabilitation and in particular for the patient user group [19–21]. However, the development of a competency model is of great importance for the successful implementation and dissemination of telerehabilitation, as it can be used to determine if the current workforce possesses the competencies necessary to successful telerehabilitation usage, to select appropriate patients and therapists for using or providing telerehabilitation according to the needed competencies, or to design a curriculum for patient and therapist training [17].

Against this background, the present study aims on the identification of required competencies and the development of a competency model for telerehabilitation therapists and patients in the context of telerehabilitation aftercare in Germany. Our study is grounded by four research questions:

- What tasks and requirements are associated with the use of telerehabilitation?
- What competencies are required by therapists and patients for a successful usage of telerehabilitation?
- How do the competencies differ regarding the target group and other person- or context-related characteristics (e.g., age, gender, affinity for technology, type of telerehabilitation, job)?
- How can the identified competencies be systematized in a competency model?

## Materials and methods

### Study design

We conducted two cross-sectional online surveys, one with telerehabilitation patients and one with telerehabilitation therapists from Germany. The surveys had two principal areas of content: on the one hand, it dealt with the work steps and required competencies in telerehabilitation, on the other hand, it evaluated the training needs regarding telerehabilitation. This article only reports on the steps and competencies. Ethical approval was granted by the University of Siegen, Germany (ER_4/2022). We follow the STrengthening the Reporting of OBservational studies in Epidemiology (STROBE) guideline, in particular the checklist for cross-sectional studies [22,23] (S1 Appendix).

We used the seven-step modeling process by Marelli et al. [17] for methodological guideline. This included the definition of objectives, gaining a sponsor, and planning how to communicate with the stakeholders involved. In step four, the methodology was planned. This online survey was based on previous results of a scoping review [16] and focus groups [24] resulting in a preliminary list of relevant competencies. The online survey served to further specify, prioritize, and systematize relevant competencies. In step five, data collections were realized and the competencies were identified and organized into a model. In step six, the competency model will be applied in order to select, train, manage, reward, or compensate employees or other affected groups. Lastly, the developing process and the resulting model will be evaluated [17].

### Recruitment process and target group

The target group were patients and therapists in Germany who had offered or used a telerehabilitation service approved in standard care or temporarily authorized by the German pension insurance (all of which are digital services for medical rehabilitation aftercare) within the past two years. We recruited both across indications (different somatic and psychosomatic illnesses) and across programs (both synchronous video consultation platforms and apps for asynchronous use of all program providers). The aim was to conduct a complete survey of all suitable patients and therapists. However, as no register of contact details for individual patients and therapists was available, all facilities with a corresponding digital service were contacted as a larger unit.

Recruitment started in May 2023. To minimize selection bias, we used different recruitment strategies. First, we searched for all rehabilitation facilities and practices that offered telerehabilitation aftercare. We identified 351 facilities via the website of the German pension insurance (https://nachderreha.de, May 2023) and, depending on available contact details, we contacted the clinic management, therapy management or those responsible for telerehabilitation by email with a request to forward the information materials to the target groups. Second, we contacted the program providers (with digital programs approved by the German pension insurance) by email. Through individual agreements with the eleven program providers and one teletherapy clinic, the information materials were distributed via newsletter, in direct contact with cooperating facilities, internal chats or directly via the telerehabilitation program. Further, we contacted 377 self-help contact centers in Germany and twelve federal or state associations in ​​rehabilitation by email with the request to forward the information materials to the target groups or to spread the surveys via their newsletter or homepage. Lastly, we posted the information flyer at relevant social media platforms (rehabilitation groups on Facebook and patient forums).

We developed information materials, including two vivid flyers (translated from German to English, S2 Appendix and S3 Appendix), an invitation letter and a detailed information sheet on the project and data protection. The material contained a QR-code and/or a link to the questionnaire. The participants gave informed consent by clicking a tick box on the starting page of the survey. All data were collected anonymously.

### Development of the questionnaires

We developed two surveys via the online survey tool Limesurvey–one for telerehabilitation patients and one for therapists. The questions were developed with regard to the “10 commandments” of question formulation by Porst [25]. The questions were based on the results of a scoping review and focus groups. Thus, relevant work steps were divided into a preparation and a realization phase. Required competencies were systematized into the components knowledge, skills, attitudes, experience, and personal/sociodemographic factors. A draft questionnaire was pretested (qualitative pretest [12]) by two scientists in digital public health and telerehabilitation, as well as by one telerehabilitation therapist and two specialists from different telerehabilitation program providers, and subsequently revised. The pre-test showed a processing time of approx. 15 minutes.

The final online surveys (translated from German to English, S4 Appendix, S5 Appendix) included primarily closed and a few open questions. We divided the questionnaires into six blocks, of which four are relevant for competency modeling: information on the telerehabilitation program used and experience with it, steps in telerehabilitation, required competencies, technology affinity (TA), and sociodemographic data. We collected the following data on the telerehabilitation program: current/past use, program type, use of wearables, indication group, duration and frequency of use, and for therapists type of therapy, type of job (only tele-therapist vs. on-site and tele-therapist), as well as type of rehabilitation facility. Regarding the steps of telerehabilitation, the participants had to select the relevant tasks from pre-formulated tasks, which could be supplemented by an open answer. It was also assessed whether the participants see themselves as responsible for the tasks conducted. With regard to the required competencies, participants were asked to assess the relevance of pre-formulated competencies divided into the components knowledge, skills, attitudes, experience (based on the definition by Marelli et al. [17] and results of a scoping review [16]). For patients, this was 26 competencies and for therapists 28. The relevance of a competency was rated along a 7-point scale, from 1 = “not at all important” to 7 = “very important” (referring to the Hennessy-Hicks Training Needs Assessment Questionnaire [26]). Regarding personal characteristics, a yes/no question was used to ask whether these can influence successful telerehabilitation usage. An open question asked about other relevant competencies. Technology affinity was determined following Franke et al. [27] (scale: 1–6, a higher number indicating a higher affinity). Lastly, we collected following sociodemographic data: gender, age, highest educational/professional qualification, and for patients, access to a social support.

### Data collection

The survey ran from June until August 13, 2023. During the 2.5 months, 272 patients filled out the questionnaire completely, 209 incompletely (43.5% drop-out). 74 therapists filled out the questionnaire completely, 50 incompletely (40.3% drop-out).

### Data preparation and statistical analysis

#### Preparation of the dataset

For data analysis, only the data sets of fully completed questionnaires were used (n_patients_ = 272; n_therapists_ = 74). Thus, missing values resulted in the exclusion of cases. To enable group comparisons, individual cases that did not provide information on age (three patients), gender (five patients; one therapist), and education (five patients) were excluded. Cases that indicated "diverse" for gender (one patient) were also excluded, as this group was too small for group comparison. This resulted in an adjusted data set of 262 patients and 73 therapists for data analysis. We used Stata 15.1 for all analyses.

#### Descriptive analyses

We used descriptive analyses to outline the characteristics of the study population and to provide the distribution of responses, e.g., regarding the relevance of competencies as perceived by the respondents. Distributions were investigated using absolute/relative frequencies, and additionally medians (Mdn) for ordinal variables, and means (M) and standard deviations (SD) for interval variables.

#### Bivariate analyses

We conducted bivariate analyses to examine whether mean scores on the relevance of competencies differ systematically between subgroups. As the dependent variable had an interval scale and the independent group variables had or were converted to a nominal scale with two independent groups, we performed independent t-tests or U-tests. The dependent variable is the relevance of each competency as rated by the respondents on a scale from 1 (not at all important) to 7 (very important). For therapists and patients, six or respectively four knowledge areas, twelve skills, seven attitudes and three experiences were queried. These competencies were combined into index variables for each competency dimension (knowledge index, skill index, attitude index, experience index). The group variables are gender, age, type of telerehabilitation program, technology affinity and job (only for therapists). To facilitate comparison, we created new dichotomous variables. To avoid a too large difference between group sizes, therapists were divided along the median age of the sample into the groups <40 and ≥40, whereas patients were divided into <50 and ≥50 (corresponding to the average age of German rehabilitants of 53 [28]). Technology affinity was measured as a mean (scale 1–6) and divided into “low” (1–3.5 points) and “high” (3.6–6 points). Table 1 describes the group variables and group sizes.

**Table 1:** Group variables and size according to target group (n_patients_ = 262, n_therapists_ = 73, adjusted data set).

	Patients (N = 262)				Therapists (N = 73)			
Group Variable	Group 1	Group 2	Size Group 1	Size Group 2	Group 1	Group 2	Size Group 1	Size Group 2
Age	<50	≥50	70	192	<40	≥40	35	38
Gender	Men	Women	62	200	Men	Women	24	49
Program	Video	App	113	149	Video	App	15	58
Technology affinity	Low	High	129	133	Low	High	29	44
Job	x	x	x	x	Only tele-therapist	On-site and tele-therapist	15	58

The prerequisites for t-tests are approximately normally distributed data and approximately equal variances in both groups. We tested this with the Shapiro-Wilk and Levene test (significance level 0.05) [29]. T-tests can be applied regardless of sample size if the assumptions made are met. Since t-tests are comparisons of means, we may expect that with large sample sizes (>30 per group) the assumption of normally distributed data is no longer critical [30]. Table 2 describes the process of selecting the statistical test. S6 Appendix shows the result of the Shapiro-Wilk and Levene test, and the chosen test.

**Table 2:** Selection of the statistical test following Bortz & Schuster [30].

Normal distribution	Equal variances	Test
Yes	Yes	T-test robust
Yes	No	Wrong decisions in t-test probably ➔ unequal variance t-test (Welch-test)
No	Yes	T-test robust if n>30 per group Wrong decisions in t-test probably if n< = 30 per group ➔ U-test
No	No	Wrong decisions in t-test probably ➔ U-test

#### Model development

Based on the results of the online surveys we developed competency models for telerehabilitation patients and therapists. The models considered the core competencies, respectively all competencies that were rated on average highly relevant by the participants (>5.0 points on a scale of 1–7) in the total sample or one of the subgroups. In case of personal characteristics, these were integrated if they were considered relevant by at least 50% of the total sample or one subgroup. The steps of telerehabilitation were considered if they were provided by at least 50% of the total sample or one of the subgroups. Furthermore, the statistically significant group variables were included. As the goal of a competency model is not to create an exhaustive list of competencies, but to develop a list of most crucial competencies for an easy, everyday application of the model [17], competencies and elements of the model were summarized, if it could be justified in terms of content and method. Skills were divided into four skill types based on the OECD Learning Compass 2030 [31,32]. These include social/emotional, practical/physical as well as cognitive/meta-cognitive skills. For a better comprehensibility and applicability of the model, the former type was subdivided into basic cognitive skills and self-regulation skills. The skill types are defined as follows. “Physical skills are a set of abilities to use physical tools, operations and functions […]. Practical skills are those required to use and manipulate materials, tools, equipment and artefacts to achieve particular outcomes” including the ability to use new information and communication technology devices. “Social and emotional skills are a set of individual capacities that can be manifested in consistent patterns of thoughts, feelings and behaviours that enable people to develop themselves, cultivate their relationships […], and exercise their […] responsibilities.” Lastly, cognitive skills are “a set of thinking strategies that enable the use of language, numbers, reasoning and acquired knowledge” [32], whereas self-regulation skills in particular are “abilities that help a person to control and monitor their own behavior, thoughts and changing flexibly them in accordance with the demands of the situation” [33].

## Results

### Sample description

272 patients and 74 patients filled out the questionnaire completely. After data adjustment, 262 patients and 73 therapists remained. The median age of patients is 50–59 years. More than three quarters are female (76.3%) and 50% have completed their A-Levels (high school diploma) or have a higher educational/professional qualification (median). There are slightly more patients with a higher technology affinity (50.8%). The mean value of affinity for technology is 3.5 (scale 1–6; SD = .0686). The majority of patients (56.5%) has no one in their social network who could support them in using telerehabilitation. The median age of therapists is 40–49 years. Approximately two thirds are female (67.1%). 50% of therapists have completed their Master’s degree or have a higher educational/professional qualification (median). There are more therapists with a higher affinity for technology (60.3%). Across all therapists, the mean value of affinity for technology is 3.9 (scale 1–6; SD = .1334) (see Table 3).

**Table 3:** Sociodemographic and personal characteristics of the sample.

		Patients		Therapists	
Variable	Value	n	%	n	%
Age					
	20–29 years	2	0.8	15	20.5
	30–39 years	23	8.8	20	27.4
	40–49 years	45	17.2	17	23.3
	50–59 years	141	53.8	14	19.2
	60–69 years	51	19.5	7	9.6
	N	262	100.0	73	100.0
Gender					
	Male	62	23.7	24	32.9
	Female	200	76.3	49	67.1
	N	262	100.0	73	100.0
Qualification					
	No educational qualifications	1	0.4	0	0.0
	“Hauptschulabschluss” (lower secondary school qualification)	5	1.9	0	0.0
	“Mittlere Reife” (secondary school certificate)	46	17.6	0	0.0
	“Fachhochschulreife” (specialized A-levels)	20	7.6	2	2.7
	“Allgemeine Hochschulreife” (A-levels)	26	9.9	3	4.1
	Vocational training	87	33.2	12	16.4
	Bachelor’s degree / equivalent educational program	30	11.5	12	16.4
	Master’s degree / equivalent educational program	43	16.4	37	50.7
	Promotion	4	1.5	7	9.6
	N	262	100.0	73	100.0
Technology affinity					
	Low (1–3.5 points)	129	49.2	29	39.7
	High (3.6–6 points)	133	50.8	44	60.3
	N	262	100.0	73	100.0
Social support					
	Yes	114	43.5	x	x
	No	148	56.5	x	x
	N	262	100.0	x	x

#### Telerehabilitation programs used and experience

The distribution of the telerehabilitation programs used differs between patients and therapists. While both types are represented in similar quantities among patients (56.9% app usage), therapists mainly have experience with programs in which patients use the app independently (79.5%). While wearables are applied in 10.7% of the programs used by patients, this percentage is twice as high for therapists (20.5%). In terms of patients, over 94% of programs used relate to psychosomatic or orthopedic illnesses. These indications are also the focus of the therapists’ used programs, but the other indication areas are also represented with up to 31.5% (multiple answers possible). The median time patients used their digital program was between three and less than six months and for therapists six month or longer. Fifty percent of patients use the telerehabilitation program once a week or more often (median), while for therapists the median frequency is several times a week. Therapists most frequently practice sports/movement therapy (54.8%), physiotherapy (39.7%), or psychotherapy (37.0%) (multiple answers possible). 79.5% of therapists work on-site in a rehabilitation facility in addition to their work as a tele-therapist. While a large proportion works in inpatient facilities (58.9%), fewer are based in outpatient practices (27.4%) or pure tele-therapy clinics (13.7%) (see Table 4).

**Table 4:** Description of the telerehabilitation programs used and experience.

		Patients		Therapists	
Variable	Value	n	%	n	%
Telerehab program					
	Independent app use by patients	149	56.9	58	79.5
	Therapist-led video conferencing	113	43.1	15	20.5
	N	262	100.0	73	100.0
Use of wearables					
	Yes	28	10.7	15	20.5
	No	234	89.3	58	79.5
	N	262	100.0	73	100.0
Indication group*					
	Psychosomatic disease	170	64.9	33	45.2
	Orthopedics	77	29.4	38	52.1
	Cardiology	9	3.4	22	30.1
	Neurology	11	4.2	23	31.5
	Oncology	14	5.3	15	20.5
	Pulmonology	2	0.8	13	17.8
	Other	16	6.1	4	5.5
	N	299*	114,1*	148*	202,7*
Duration of use					
	Less than 1month	27	10.3	1	1.4
	Between 1 and less than 3 months	91	34.7	6	8.2
	Between 3 and under 6 months	90	34.4	10	13.7
	6 months or longer	54	20.6	56	76.7
	N	262	100.0	73	100.0
Frequency of use					
	Once a month or less	9	3.4	3	4.1
	Several times a month	22	8.4	7	9.6
	Once a week	110	42.0	12	16.4
	Several times a week	86	32.8	29	39.7
	Daily	35	13.4	22	30.1
	N	262	100.0	73	100.0
Type of therapy*					
	Psychotherapy	x	x	27	37.0
	Physiotherapy	x	x	29	39.7
	Sports/movement therapy	x	x	40	54.8
	Speech therapy	x	x	2	2.7
	Occupational therapy	x	x	9	12.3
	Other	x	x	6	8.2
	N	x	x	113*	154,7*
Type of job					
	Only tele-therapist	x	x	15	20.5
	Tele-therapist and on-site therapist	x	x	58	79.5
	N	x	x	73	100.0
Type of rehab facility					
	Tele-Therapy Clinic	x	x	10	13.7
	Inpatient rehabilitation facility	x	x	43	58.9
	Outpatient practice	x	x	20	27.4
	N	x	x	73	100.0

* Multiple answers possible. Therefore, the total number exceeds 262 or 73.

#### Steps in telerehabilitation

Participants could choose from five given answers which steps they had gone through to prepare for telerehabilitation and from eight given answers which steps they had to carry out during the execution of telerehabilitation. Regarding the patient sample, the only step the majority completed is the independent information about the program (52.7%). Participation in a personal conversation or presentation with information about the program was realized by almost half (48.9%, subgroup app users comprises 51.7%). Whereas individual adaptation of the therapy to own needs (34.7%), participation in a practical introduction (27.5%) and technical setup (19.1%) were less frequently undergone. 9.5% of patients added steps for preparation in the open text field. These were largely specifications of the listed steps, such as conversation via telephone with the therapist, on-site consultation with the social services, or test runs and accompanying training on-site.

In terms of tasks during digital therapy, “Following the therapist’s instructions” and “Motivating oneself” were performed by approximately two thirds of patients (70.2%, 63.0%). In addition, around half of the patients reminded themselves (50.0%) and adapted the therapy to individual needs (e.g., selecting content, difficulty) (51.5%). The following tasks were less relevant during execution of telerehabilitation: dealing with health problems (e.g., certain symptoms or emergencies) (39.3%, subgroup video users comprises 54.9%), self-monitoring (e.g., therapy progress, health parameters) (31.3%), documentation of therapy sessions (16.8%) and solving technical problems (16.0%). 9.2% of patients added steps relevant during telerehabilitation in the open text field. These were largely specifications of the listed steps, such as writing down exercises or journaling as documentation. One step not previously listed is the communication with other rehabilitation patients or therapists about the program, e.g., via chat.

Overall, one third of the patients (34.4%) saw their therapists or other persons as responsible for some of the listed tasks. The open text responses show that this applies to the adaptation to individual needs (26 answers), solving technical problems (10 answers) and motivation (7 answers) in particular.

The analysis shows different results for the therapists. The majority completed four out of five of the steps listed for preparation. These are: informing patients about the program (90.4%), individual adaptation of the therapy to patient’s needs (86.3%), practical instruction of patients to the program (74.0%) and supporting patients with the technical setup (72.6%). The own technical setup of the program was conducted less frequently (37.0%, subgroup male therapists comprises 54.2%). 13.7% of therapists added steps for preparation in the open text field. These were partly repetitions of the listed tasks or as a new step bureaucratic tasks (e.g., setting up patients in the program, filling out forms).

During digital therapy, most therapists conducted all listed tasks. Nearly all therapists adapted the therapy to the patient’s needs (86.3%). About three quarters guided patients through the digital therapy (75.3%) and provided feedback or motivation (74.0%). About two thirds reminded patients (69.9%), supported them with health problems (65.8%) or monitored them (e.g., therapy progress, health parameters; 65.8%). Lastly, more than half documented therapy sessions (56.2%) or solved technical problems (52.1%). 13.3% of therapists added steps relevant during telerehabilitation in the open text field. These were e.g., creating crisis plans for psychosomatic patients or having coaching phone calls with patients. Others explained that–as on-site therapists–they are only involved in preparing patients for telerehabilitation and that external providers conduct the provision of digital therapy.

One quarter of therapists (24.7%) saw their patients or other persons as responsible for some of the listed tasks. E.g., the technical support mentioned in the open text responses stood out (6 answers).

#### Required competencies for telerehabilitation

Participants rated on a scale of 1–7 how relevant they consider competencies for the successful practice of telerehabilitation. The following Tables 5 and 6 show the top five competencies, including means (M) and standard deviations (SD) for all patients/therapists separated by type of program and, for therapists, by type of job (see S8 Appendix for all variables).

**Table 5:** Top five relevant competencies for telerehabilitation patients.

	Patients (all, n = 262)	Patients (video user, n = 113)	Patients (app user, n = 149)
1	Self-interest in program (M = 6.3, SD = .0661)	Self-interest in program (M = 6.2, SD = .1005)	Self-interest in program (M = 6.4, SD = .0875)
2	Self-awareness (M = 5.9, SD = .0797)	Communication skills (M = 6.0, SD = .1166)	Self-management (M = 6.2, SD = .0909)
3	Self-management (M = 5.8, SD = .0823)	Self-awareness (M = 5.9, SD = .1211)	Willingness to learn (M = 6.0, SD = .1030)
4	Open-mindedness (M = 5.8, SD = .0773)	Reflectivity (M = 5.8, SD = .1235)	Open-mindedness (M = 5.9, SD = .1050)
5	Motivational skills (M = 5.7, SD = .0851)	Empathy (M = 5.7, SD = .1287)	Motivational skills (M = 5.9, SD = .1108)

**Table 6:** Top five relevant competencies for telerehabilitation therapists.

	Therapists (all, n = 73)	Therapists (video user, n = 15)	Therapists (app user, n = 58)	Therapists (tele, n = 15)	Therapists (on-site, n = 58)
1	Therapeutic-professional skills (M = 6.3, SD = .1725)	Medical Knowledge (M = 6.4, SD = .2895)	Therapeutic-professional skills (M = 6.3, SD = .1914)	Empathy (M = 6.8, SD = .1447)	Therapeutic-professional skills (M = 6.3, SD = .2138)
2	Medical knowledge (M = 6.1, SD = .1650)	Therapeutic-professional skills (M = 6.3, SD = .4102)	Telerehab knowledge (M = 6.1, SD = .2064)	Communication skills/ Therapeutic-professional skills (M = 6.7, SD = .1869/ .1260)	Medical knowledge (M = 6.0, SD = .1982)
3	Telerehab knowledge (M = 6.1, SD = .1870)	Communication skills/Adaptability (M = 6.1, SD = .4079/ .3960)	Medical knowledge (M = 6.0, SD = .1936)	Motivational skills/ Medical knowledge (M = 6.4, SD = .2545/ .2350)	Telerehab knowledge (M = 6.0, SD = .2179)
4	Open-mindedness (M = 6.0, SD = .1711)	Empathy (M = 6.0, SD = .4781)	Open-mindedness (M = 6.0, SD = .1944)	Telerehab knowledge (M = 6.3, SD = .3473)	Open-mindedness (M = 5.9, SD = .2018)
5	Empathy (M = 5.9, SD = .1894)	Open-mindedness (M = 5.8, SD = .3677)	Self-management/ Willing to learn (M = 5.8, SD = .2025/ .1980)	Technology acceptance/ Willing to learn (M = 6.3, SD = .2840/.2667)	Self-management (M = 5.7, SD = .2180)

Patients rated 80.8% of all competencies as highly relevant (score of 5.1 or higher). Regarding the competency components, patients rated having the right attitude (index variable, M = 5.5, SD = .0628) and the right skills (index variable, M = 5.5, SD = .0674) as most important. The most relevant individual competencies were self-interest in the program (M = 6.3, SD = .0661), self-awareness (M = 5.9, SD = .0797) and self-management (M = 5.8, SD = .0823). The least relevant competencies were experience with digital health apps (M = 3.5, SD = .1177) and experience with analogue therapy (M = 3.9, SD = .1262). Looking at the relevancies separated by the type of program, differences become apparent. The most relevant competency for video conference users was–besides self-interest in the program (M = 6.2, SD = .1005) and self-awareness (M = 5.9, SD = .1211)–communication skills (M = 6.0, SD = .1166). The most relevant competency for app users was–besides self-interest in the program (M = 6.4, SD = .0875) and self-management (M = 6.2, SD = .0909)–willingness to learn (M = 6.0, SD = .1030).

Therapists rated 78.6% of all competencies as highly relevant. Regarding the competency components, therapists rated having the right skills (index variable, M = 5.5, SD = .1545) and the right attitude (index variable, M = 5.4, SD = .1458) as most important. The most relevant individual competencies were therapeutic-professional skills (M = 6.3, SD = .1725), medical knowledge (M = 6.1, SD = .1650) and telerehabilitation knowledge (M = 6.1, SD = .1870). The least relevant competencies were experience with digital work apps (M = 4.3, SD = .1961) and process knowledge (M = 4.4, SD = .1897). The most relevant competencies for therapists guiding video conferences were–next to therapeutic-professional skills (M = 6.3, SD = .4102) and medical knowledge (M = 6.4, SD = .2895)–communication skills and adaptability (M = 6.1, SD = .4079, SD = .3960). Whereas the three most relevant competencies accessed by therapists supervising app therapy are the same than by the total sample. The most relevant competencies for tele-therapists were–next to therapeutic-professional skills (M = 6.7, SD = .1260) and medical knowledge (M = 6.4, SD = .2350)–empathy (M = 6.8, SD = .1447), communication skills (M = 6.7, SD = .1869) and motivational skills (M = 6.4, SD = .2545). The three most important competencies of the subgroup “therapists working on-site in facilities” match the overall sample of therapists.

Table 7 displays participants’ responses regarding the influence of personal characteristics on the successful use of telerehabilitation. Patients found that gender (11.5% yes), place of residence (17.6% yes) and socioeconomic status (27.9% yes) tend to have no influence on successful use of the program, but they were divided on the level of education (42.0% yes, 58.0% no). Most patients saw an influence from age (56.9% yes), severity of their own illness (63.7% yes) and language abilities (67.6% yes). Therapists tended to see no influence for gender (5.5% yes), socioeconomic status (16.4% yes) and level of education (38.4% yes), but they were split on age (48,0% yes, 52.0% no). Most therapists saw an influence from type of therapy practiced (53.4% yes) and language abilities (71.2% yes).

**Table 7:** Influence of personal characteristics on telerehabilitation use.

	Patients, answering “Yes”		Therapists, answering “Yes”	
Variable influencing successful usage	n	%	n	%
Age	149	56.9	35	48.0
Gender	30	11.5	4	5.5
Level of education	110	42.0	28	38.4
Language	177	67.6	52	71.2
Socioeconomic status	73	27.9	12	16.4
Residence (urban vs. rural)	46	17.6	X	X
Severity of the disease	167	63.7	X	X
Type of therapy practiced	X	X	39	53.4

#### Group differences

We analyzed if mean scores on the relevance of competencies differ systematically between subgroups. Table 8 shows the competency indices analyzed by group variables, statistical test conducted and p-value (a corresponding overview for each individual competency can be found in S9 Appendix).

**Table 8:** Group differences regarding the relevance of competency indices.

		Patients		Therapists	
Competency	Group variable	Test	P-value	Test	P-value
Knowledge index					
	Age	T-Test	.1446	U-Test	.9558
	Gender	T-Test	.5862	U-Test	.7639
	Program	T-Test	.1529	U-Test	.5242
	Technology affinity	T-Test	.2753	U-Test	.9955
	Job	x	x	U-Test	.9836
Skill index					
	Age	T-Test	.0479*	U-Test	.8901
	Gender	T-Test	.9975	U-Test	.5370
	Program	T-Test	.7969	U-Test	.5249
	Technology affinity	T-Test	.0710	U-Test	.8478
	Job	x	x	U-Test	.5845
Attitude index					
	Age	T-Test	.4948	U-Test	.2475
	Gender	T-Test	.4082	U-Test	.7731
	Program	T-Test	.0084*	U-Test	.4156
	Technology affinity	T-Test	.1392	U-Test	.7776
	Job	x	x	U-Test	.7067
Experience index					
	Age	T-Test	.4723	U-Test	.3363
	Gender	T-Test	.0635	U-Test	.7451
	Program	T-Test	.0064*	U-Test	.3904
	Technology affinity	T-Test	.0320*	U-Test	.0920
	Job	x	x	U-Test	.4706

* Significant result

Various personal characteristics influence the assessment of the relevance of telerehabilitation competencies. Looking at the sample of patients, the type of telerehabilitation program used has a significant influence on almost half of the competencies. Thus, patients using a therapeutic app independently rate the following competencies more important: attitudes in general (index variable, M_video_ = 5.3, M_app_ = 5.6, p = .0084), experiences in general (index variable, M_video_ = 3.9, M_app_ = 4.4, p = .0064), telerehabilitation knowledge (M_video_ = 5.1, M_app_ = 5.6, p = .0143), medical knowledge (M_video_ = 5.2, M_app_ = 5.6, p = .0357), motivational skills (M_video_ = 5.5, M_app_ = 5.9, p = .0328), self-management skills (M_video_ = 5.4, M_app_ = 6.2, p = .0000), reading/writing skills (M_video_ = 4.6, M_app_ = 5.5, p = .0001), technology affinity (M_video_ = 4.4, M_app_ = 4.9, p = .0038), willingness to learn (M_video_ = 5.5, M_app_ = 6.0, p = .0020), self-efficacy expectation (M_video_ = 5.1, M_app_ = 5.5, p = .0320), and experience with digital health apps (M_video_ = 3.1, M_app_ = 3.9, p = .0009). Whereas patients using therapist-led video conferencing rated empathy (M_video_ = 5.7, M_app_ = 5.1, p = .0223) and teamwork skills (M_video_ = 5.7, M_app_ = 5.0, p = .0321) more important. Furthermore, technology affinity has an influence. Patients with a higher technology affinity (3.6–6 points) attributed higher relevance to experiences in general (index variable, M_lowTA_ = 4.0, M_highTA_ = 4.4, p = .0320), legal knowledge (M_lowTA_ = 4.8, M_highTA_ = 5.2, p = .0402), patience (M_lowTA_ = 5.2, M_highTA_ = 5.8, p = .0061), experience with digital health apps (M_lowTA_ = 3.2, M_highTA_ = 3.8, p = .0093) and open-mindedness (MlowTA = 5.6,MhighTA = 6.0, p = .0239). Older patients (≥50 years) rated skills in general as more relevant (index variable, M_younger_ = 5.3, M_older_ = 5.6, p = .0479), as well as adaptability (M_younger_ = 5.0, M_older_ = 5.5, p = .0088) and teamwork skills (M_younger_ = 4.8, M_older_ = 5.5, p = .0152). Lastly, male patients attributed higher relevance to experience in analogue therapy (M_female_ = 3.7, M_male_ = 4.5, p = .0066).

Looking at the sample of therapists, fewer significant differences become apparent. Table 8 shows no significant group differences regarding the competency indices. Considering individual skills, there were significant differences in terms of age, affinity for technology, and job type. Thus, younger therapists (<40 years) rated knowledge about telerehabilitation (M_younger_ = 6.5, M_older_ = 5.6, p = .0094), technology-related knowledge (M_younger_ = 5.2, M_older_ = 4.2, p = .0171), teamwork skills (M_younger_ = 5.1, M_older_ = 4.2, p = .0391) and technology acceptance as more relevant (M_younger_ = 6.0, M_older_ = 5.3, p = .0279). Therapists with a lower technology affinity (1–3.5 points) attributed higher relevance to legal (M_lowTA_ = 5.6, M_highTA_ = 4.8, p = .0418) and medical knowledge (M_lowTA_ = 6.6, M_highTA_ = 5.8, p = .0231) as well as experience with digital devices (MlowTA = 5.6,MhighTA = 4.9, p = .0423), whereas therapists with a higher technology affinity (3.6–6 points) rated self-interest in the program (M_lowTA_ = 4.9, M_highTA_ = 5.4, p = .0211) as more relevant. Lastly, therapists that work exclusively as tele-therapists consider empathy (M_tele_ = 6.8, M_on-site_ = 5.6, p = .0026), communication skills (M_tele_ = 6.7, M_on-site_ = 5.6, p = .0100) and technology acceptance (M_tele_ = 6.3, M_on-site_ = 5.5, p = .0216) more relevant than therapists that work as well on-site. We did not identify significant differences for gender or program type.

#### Competency models

Based on the results, we developed the competency models for telerehabilitation for the target group of patients and therapists. The models describe the core competencies and possible determinants (personal characteristics as “enabling factors”) relevant for a successful execution of the core steps in telerehabilitation (with focus on telerehabilitation aftercare).

**Fig 1:**
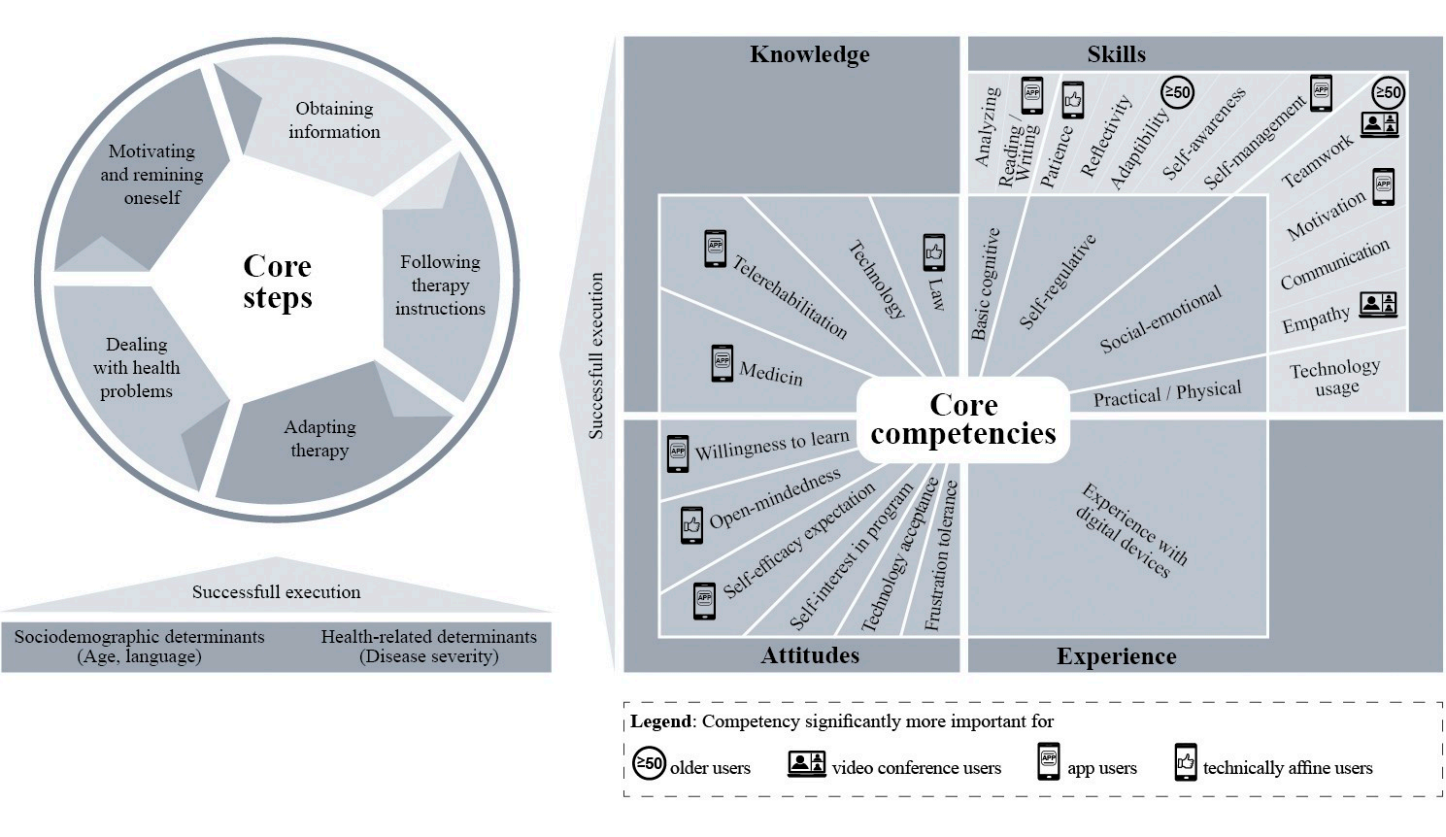
Competency model for telerehabilitation patients.

Beginning with the competency model for patients (see Fig 1), the tasks patients execute in the context of telerehabilitation programs can be grouped into five core steps. These include obtaining information about the program in preparation of its usage, following the therapist’s instructions while exercising, adapting the therapy to one’s own individual needs (e.g. selecting content, difficulty levels), independently dealing with health problems (e.g. in case certain symptoms or medical emergencies occur), as well as motivating and reminding oneself to execute the digital therapy.

For patients, 23 core competencies are relevant for a successful execution of telerehabilitation programs, including four knowledge areas, six attitudes, one field of experience and twelve skills which are subdivided into four skill types for better manageability and usability. Accordingly, patients need medical, telerehabilitation-related, technology-related, and legal knowledge (for video conferencing) for successful program usage. Required is also a positive attitude including self-interest in the program, open-mindedness, willingness to learn, a sufficient self-efficacy expectation, technology acceptance and frustration tolerance. Also, experience in using digital devices is necessary (for app users). Relevant self-regulation skills include patience, self-awareness, self-management, reflectivity and adaptivity. Basic cognitive skills comprise reading/writing skills and analytic skills. Required social and emotional skills are empathy, teamwork, motivation and communication skills. Lastly, practical, and physical skills that are necessary are skills in technology usage.

Other personal characteristics of patients can enable the successful execution of telerehabilitation tasks. These are health-related determinants (disease severity) and sociodemographic determinants (age, language abilities).

Several factors influence the perceived relevance of competencies by patients. Most important is the type of telerehabilitation program. Thus, patients independently using an app rate telerehabilitation and medical knowledge, motivational and self-management skills, reading/writing skills, willingness to learn and self-efficacy expectation significantly more relevant. Whereas patients using therapist-led video conferencing rate empathy and teamwork skills significantly more important. Patients with a higher technology affinity attribute significantly higher relevance to legal knowledge, patience, and open-mindedness. Older patients consider adaptability and teamwork skills significantly more relevant.

**Fig 2:**
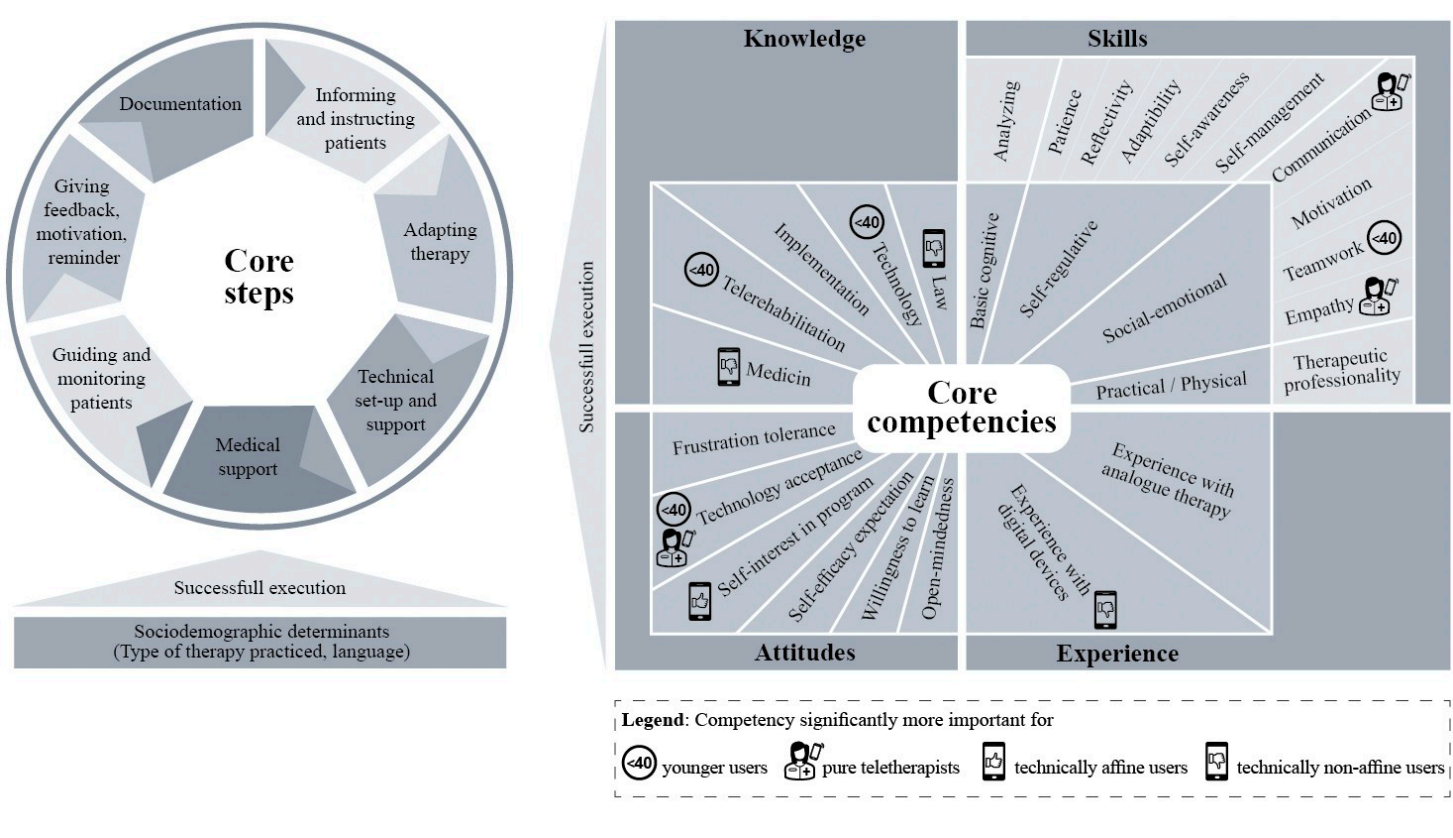
Competency model for telerehabilitation therapists.

Regarding the competency model for therapists (see Fig 2), the work tasks required for telerehabilitation can be compiled into seven core steps. These include informing and practically instructing patients in preparation of program usage, adapting the therapy to the patient’s needs before and during digital therapy, carrying out technical set-up and support, giving medical support, guiding and monitoring the patient’s therapy execution (e.g. therapy progress or health parameters), giving feedback, motivation and reminder to the patient, and lastly documenting the digital therapy sessions.

For therapists, 24 core competencies are relevant for a successful execution of telerehabilitation programs, including five knowledge areas, six attitudes, two fields of experience and eleven skills which are subdivided into four skill types for better manageability and usability. Accordingly, therapists need medical, telerehabilitation-related, implementation-related, legal, and technology-related (for tele-therapists) knowledge for successful program usage. Required is also a positive attitude including open-mindedness, willingness to learn, technology acceptance, a sufficient self-efficacy expectation, self-interest in the program, and frustration tolerance. Also, experience in analogue therapy and in using digital devices is necessary. Relevant self-regulation skills include self-management, patience, self-awareness, adaptivity, and reflectivity. A relevant basic cognitive skill are analytic skills. Required social and emotional skills are empathy, motivation skills, communication skills and teamwork skills (for tele-therapists). Lastly, necessary practical and physical skills are therapeutic-professional skills to carry out therapy properly.

Other personal characteristics of therapists can enable the successful execution of telerehabilitation tasks, comprising different sociodemographic determinants (form of therapy practiced, language abilities).

Several factors influence the perceived relevance of competencies by therapists. Most important is the age of therapists. Thus, younger therapists rate knowledge about telerehabilitation, technology-related knowledge, teamwork skills and technology acceptance as more relevant than older therapists. Therapists with a lower technology affinity attribute higher relevance to legal and medical knowledge as well as experience with digital devices, whereas therapists with a higher technology affinity rate self-interest in the program as more relevant. Lastly, therapists that work exclusively as tele-therapists consider empathy, communication skills and technology acceptance more relevant than therapists that work as well on-site in facilities.

## Discussion

### Principal findings

In addition to the actual execution of digital therapy, telerehabilitation places further requirements on the users. For example, patients must be able to independently gather information about the program, adapt the digital therapy, respond to health problems and motivate and remind themselves. Therapists must interact with patients and for example inform, instruct, motivate and remind them via digital devices. At the same time, they are responsible for the technical realization and for managing health problems remotely. This requires a wide range of competencies, which can be systematized in a competency model with 23 or respectively 24 core competencies. The three most relevant competencies for patients are self-interest in the program, self-awareness and self-management. For therapists these are therapeutic-professional skills, medical and telerehabilitation knowledge. How relevant users rate the required competencies differs significantly according to age and technology affinity. Only in the group of patients do gender and the program type also have an influence. Whereas with therapists, the type of job (exclusively tele-therapist or also on-site) makes a difference.

### Comparison with prior work

A comparison of the two target groups reveals the following similarities and differences. While skills in general (index variable) are rated by patients and therapists both as the most relevant competency domain and equally important on average, patients rate knowledge in general (index variable) slightly less important (M_patients_ = 5.2 vs. M_therapists_ = 5.3), but attitudes in general (index variable) slightly more important (M_patients_ = 5.5 vs. M_therapists_ = 5.4). The greatest difference is seen in the required experiences. Here the difference in relevance is almost one point value (M_patients_ = 4.2 vs. M_therapists_ = 5.0). A higher rating of experience by therapists can be seen in all three associated competencies. The biggest difference is in “experience with analog therapy” (difference in the mean value of 1.7 points). This is also reflected in the fact that the “therapeutic-professional skills” are the most relevant competency for therapists. For these can be strengthened primarily in the acquisition and execution of analog therapy or rather the day-to-day clinical practice. Individual studies confirm that video conferencing in rehabilitation, for example, is also accepted and used by older patients who are inexperienced in this area [34]. And that prior years of clinical experience can contribute to healthcare professionals being able to provide competent telerehabilitative services [35]. However, further research is required here.

It can be seen that social-emotional skills are particularly relevant for video conference users and pure tele-therapists. Thus, in contrast to the overall sample, communication skills and empathy are among the top 5 competencies for both patients and therapists. In addition, the statistical tests show that patients who use video conferencing rate empathy and teamwork skills as significantly more important than app users, and that pure tele-therapists rate empathy and communication skills as significantly more important than therapists who also work on-site. This is in line with existing curricula for telemedicine personnel, which describe communication skills as essential [36–38]. For example, in their requirements profile for non-medical assistants in telemedicine centers in Germany, Helms et al. define social skills as one of four relevant competency domains. These include the competencies communication skills, empathy and motivational skills which we also identified. Social skills that have not been identified as core competencies for therapists in telerehabilitation, but which Helm et al. highlight, are politeness, assertiveness and friendliness [38]. A recent scoping review concludes that the required social skills of telerehabilitation patients comprise skills in remote communication taking into account the special features of video conferencing, but highlights the need for more research [16].

For users who execute or respectively supervise a telerehabilitation app, a positive attitude is particularly important. In addition to open-mindedness (and self-interest in the program among patients), willingness to learn is among the top 5 competencies among patients and therapists in contrast to the overall sample. Additionally, patients who use an app independently rather than a therapist-led video conference rate the attitudes “self-efficacy expectation” and “willingness to learn” significantly more important. These results are consistent with a qualitative online survey, which shows that telemedicine nurses should have an open attitude towards new things and a willingness to learn [39].

A look at the skills required by patients shows that self-regulative skills in particular are highly relevant (four competencies ≥5.5 points). Therefrom self-awareness is most relevant for video conference users (in third place) and self-management for app users (in second place). App users also rated self-management and (the closely related concepts of [40,41]) self-efficacy expectations and motivation as significantly more relevant than app users. We here see a transfer of responsibility from the healthcare professional to the patient in the sense of required active participation and self-care, which raises new ethical and practical questions in providing rehabilitative care [42].

Of the competencies included in the competency model, “Legal knowledge” and “Experience with digital tools” are the least important for patients. The competencies "Technology affinity", "Experience in analog therapy", and "Experience with digital health apps" did not make it into the model due to a lower relevance rating. The last two are even below mid-scale and therefore closer to the "not at all important" answer. For therapists, the competencies “Legal knowledge”, “Technology knowledge” and “Teamwork” are rated lowest in the model. The competencies "Process knowledge", "Experience with digital work apps", "Technology affinity" as well as “Technology skills” did not make it into the model due to a lower relevance rating. It is striking here that experience and technology-related skills in particular were rated as less relevant. This is consistent with the preliminary results of our focus group discussions with patients and therapists that technical competencies are rather inferior and that the digital programs for telerehabilitation aftercare are rather easy to use [24,43]. At the same time, patients in Germany usually receive an introduction to the digital program on-site, which means that only a limited amount of digital expertise may be required [44,45].

The study shows from a user perspective which competencies are considered relevant for the use of telerehabilitation. It remains unclear whether and to what extent these competencies actually have an influence on the successful usage. However, the results can be partially supported by implementation research that has been carried out. Some of the identified competencies can therefore also be found in large-scale reviews that examine the determinants for the successful implementation of telemedicine. For example, Broens et al. [46] show, in line with the present study results, that technology acceptance and the attitudes towards telemedicine influence successful implementation. Scott Kruse et al. [47] also show that certain missing skills (e.g. technical skills or eHealth literacy), negative attitudes (e.g. resistance to change) and lack of knowledge (e.g. about telehealth) hinder successful implementation.

The majority of patients in this study stated that health-related factors (such as the severity of the illness) and sociodemographic factors (such as age and language abilities) can influence the successful use of telerehabilitation. Among therapists, various sociodemographic factors (such as the type of therapy practiced and language abilities) were also considered relevant by the majority. Further studies show that the use of telemedicine might correlate with age or language abilities [48,49], and that age can be a barrier to the successful implementation of digital services [47].

### Future directions

The model was developed based on the experiences of German patients and therapists with telerehabilitation aftercare services. It now needs to be tested and validated for other countries with different rehabilitative care structures. The model is an innovation, particularly for the patient user group, in that it differentiates user competencies for digital health applications for the first time. Thus, the model can serve as a basis for identifying user competencies for other digital services in eHealth or mHealth. The study revealed that the majority of patients and therapists in the present sample assume that health-related and sociodemographic factors influence successful telerehabilitation use. Further research is needed to determine the extent to which these factors actually have an influence.

In general, the provision of telerehabilitation should continue to be ensured and expanded. By promoting telerehabilitation in addition to analog rehabilitation, a hybrid structure can be created that enables all people to make use of rehabilitative services according to their needs and competencies. In order not to exclude or disadvantage any particular population groups and to provide all patients with equal access to high-quality rehabilitation, political decision-makers should also promote the expansion of the technological infrastructure (especially in rural areas). At the same time, insurance providers and professional associations must conceptually anchor the requirements placed on users by new digital services in order to provide a transparent orientation framework for those involved in telerehabilitation.

To avoid or contain the digital divide, it is necessary to train healthcare professionals and users of telerehabilitation in order to enable them to use telerehabilitation competently and independently. Patients should be supported in the preparation, use and follow-up of telerehabilitation, for example by offering support (on-site, by telephone or online) as well as options to borrow digital devices. Patients and therapists should receive comprehensive training before and during the use of the program [50]–therapists already during their vocational training [51,52]. The competency model developed is suitable as an orientation framework for the development of such a training concept. The model’s design simplifies the promotion of competencies, as the various competency domains such as knowledge, attitudes and skills can be addressed in a differentiated manner and different teaching methods can be applied, e.g. to promote social-emotional or practical skills. In addition, suitable personnel can be recruited and employed for telerehabilitation based on the competency profile. To this end, the required core competencies should already be communicated in job advertisements. At the same time, therapists and patients can use the competency models to jointly decide which form of rehabilitation is suitable. The transparent presentation of the required competencies can enable patients to make an informed decision about the upcoming rehabilitation–based on their wishes, needs and competencies.

### Strengths and limitations

The present study is the first to focus on the patient perspective in competency modeling regarding the use of new digital health services and thus represents an innovation. As only participants who had actually used telerehabilitation were included in the surveys, it can be assumed that the results have a high internal validity. The use of standardized questionnaires with clearly formulated questions and a fixed ordering of questions increases the reliability of the results.

There are some limitations that need to be considered. First, several factors could have led to a sampling bias. For example, the patients and partly the therapists could not be contacted directly by the research team due to a lack of contact details. Instead, the information material had to be forwarded by the rehabilitation facilities, providers or associations. The clinic or therapy managers may have selected patients or therapists who were particularly interested in technology or research. In addition, participation in the survey was voluntary, so that people with a greater affinity for technology might have been more likely to respond to the survey as part of the self-selection process. However, this sampling bias could have been limited by the fact that only people who had already chosen digital therapy and used telerehabilitation took part in the surveys [53,54].

Further, certain circumstances may limit the generalizability of the results. The samples have certain characteristics that may not be transferable to the general population of telerehabilitation users. For example, the study did not include any people over the age of 70. In addition, the samples are characterized by an overrepresentation of women and highly educated people. At the same time, the results are based on data from German patients and therapists who have used telerehabilitation aftercare (as one form of telerehabilitation). As already described, the rehabilitative care systems and digital services differ between different countries, meaning that applicability may also be limited here. As there was no pretest with patients due to a lack of feedback, it is possible that patients did not understand or misinterpreted individual questions. The handling of missing values in the statistical analysis (“listwise deletion”) may also have led to inaccurate results, as it is possible that a data omission correlates with certain variables and that the subsample of complete cases differs substantially from the total number of cases surveyed and thus also from the general population [55].

Finally, it must be mentioned that this is a cross-sectional survey, which means that no causal relations or impacts could be determined. Thus, the participants were asked about their opinions of the required competencies. These assessments may have been influenced by the experiences and perceptions of the collective or subgroups. For example, it cannot be explained why older patients in our sample rated teamwork skills as more important than younger patients. Objective methods or tests must be used to find out whether the identified competencies actually facilitate successful use and which factors influence the required competencies.

## Conclusion

Research and practice have shown that a lack of user skills and literacies is a barrier to successful telerehabilitation. With the development of the competency models in this study, the relevant requirements and core competencies for the successful execution of telerehabilitation by patients and therapists were differentiated and systematized. By using these models, needs-based training can be provided in future and people can be better supported in their use. This can help to better exploit the benefits that telerehabilitation offers and reduce disparities in the provision of these services.

## Supporting information

S1 Appendix STROBE checklist. (PDF)

S2 Appendix Flyer for recruitment of patients. (PDF)

S3 Appendix Flyer for recruitment of therapists. (PDF)

S4 Appendix Online survey for patients. (PDF)

S5 Appendix Online survey for therapists. (PDF)

S6 Appendix Results of the Shapiro-Wilk- and Levene-Tests. (PDF)

S7 Appendix Steps in Telerehabilitation. (PDF)

S8 Appendix Relevance of competencies. (PDF)

S9 Appendix Results of t-tests and U-tests. (PDF)
