# Supplementary material for: Developing a competency model for telerehabilitation therapists and patients: Results of a cross-sectional online survey
Source: PLOS Digit Health. 2025 Jan 3;4(1):e0000710. doi: 10.1371/journal.pdig.0000710 (PMC11698311; doi:10.1371/journal.pdig.0000710)
Supplement: S2 Appendix — (PDF) [file pdig.0000710.s002.pdf]

Survey of patients on

COMPETENCIES AND TRAINING NEEDS IN TELEREHABILITATION AFTERCARE

Do you use telerehabilitation aftercare or have you used it?

Then we look forward to your participation in our online survey by 13.08.23.

We are looking for:

- Patients who are currently using a telerehabilitation aftercare program or have used one in the past two years

We are interested in:

- what experiences you have made
- what requirements the program has for usage
- how you have been prepared for usage

The following link will take you to the survey:

https://umfragen.uni-siegen.de/index.php/715987?lang=de

Or you can scan the QR code:

The online survey takes approx. 15 minutes and is anonymous

We are happy to answer any questions:
Stephan.Krayter@uni-siegen.de
Lea.Stark@uni-siegen.de
