# Supplementary material for: Developing a competency model for telerehabilitation therapists and patients: Results of a cross-sectional online survey
Source: PLOS Digit Health. 2025 Jan 3;4(1):e0000710. doi: 10.1371/journal.pdig.0000710 (PMC11698311; doi:10.1371/journal.pdig.0000710)
Supplement: S6 Appendix — (PDF) [file pdig.0000710.s006.pdf]

S6 appendix: Results of Shapiro-Wilk- and Levene-Tests

Table S6a: Results of Shapiro-Wilk- and Levene-Tests and corresponding selection of the statistical test regarding the relevance of competency indices by groups

		Patients			Therapists		
Competency	Group Variable	Normal Distribution	Equal variances	Statistical test	Normal Distribution	Equal variances	Statistical test
Knowledge Index							
	Age	No	Yes	T-Test*	No	No	U-Test
	Gender	No	Yes	T-Test*	No	Yes	U-Test**
	Program	No	Yes	T-Test*	No	Yes	U-Test**
	Tech. Affinity	No	Yes	T-Test*	No	Yes	U-Test**
	Job	x	x	x	No	Yes	U-Test**
Skill Index							
	Age	No	Yes	T-Test*	No	No	U-Test
	Gender	No	Yes	T-Test*	No	Yes	U-Test**
	Program	No	Yes	T-Test*	No	Yes	U-Test**
	Tech. Affinity	No	Yes	T-Test*	No	Yes	U-Test**
	Job	x	x	x	No	Yes	U-Test**
Attitude Index							
	Age	No	Yes	T-Test*	No	No	U-Test
	Gender	No	Yes	T-Test*	No	Yes	U-Test**
	Program	No	Yes	T-Test*	No	Yes	U-Test**
	Tech. Affinity	No	Yes	T-Test*	No	Yes	U-Test**
	Job	x	x	x	No	Yes	U-Test**
Experience Index							
	Age	Yes	Yes	T-Test	No	No	U-Test
	Gender	Yes	Yes	T-Test	No	No	U-Test
	Program	No	Yes	T-Test*	No	Yes	U-Test**
	Tech. Affinity	Yes	Yes	T-Test	No	No	U-Test
	Job	x	x	x	No	No	U-Test

* T-test applicable because sizes of group 1 and 2 > 30

** T-test not applicable, because sizes of group 1 and 2 <=30

Table S6b: Results of Shapiro-Wilk- and Levene-Tests and corresponding selection of the statistical test regarding the relevance of competencies by groups (Part 1)

		**Patients**			**Therapists**		
Competency	Group Variable	Normal Distribution	Equal variances	Statistical test	Normal Distribution	Equal variances	Statistical test
Telerehab. Knowlege							
	Age	No	Yes	T-Test*	No	No	U-test
	Gender	No	Yes	T-Test*	No	Yes	U-Test**
	Program	No	Yes	T-Test*	No	Yes	U-Test**
	Techn. Affinity	No	Yes	T-Test*	No	Yes	U-Test**
	Job	X	X	X	No	Yes	U-Test**
Legal Knowledge							
	Age	N	Yes	T-Test*	No	Yes	T-Test*
	Gender	No	Yes	T-Test*	No	Yes	U-Test**
	Program	No	Yes	T-Test*	No	Yes	U-Test**
	Techn. Affinity	No	Yes	T-Test*	Yes	No	Welch-Test
	Job	X	X	X	No	Yes	U-Test**
Technology Knowledge							
	Age	No	Yes	T-Test*	No	No	U-test
	Gender	No	Yes	T-Test*	Yes	Yes	T-test
	Program	No	Yes	T-Test*	Yes	Yes	T-test
	Techn. Affinity	No	Yes	T-Test*	No	Yes	U-Test**
	Job	X	X	X	Yes	Yes	T-test
Medical Knowledge							
	Age	No	Yes	T-Test*	No	No	U-test
	Gender	No	Yes	T-Test*	No	Yes	U-Test**
	Program	No	No	U-Test	No	Yes	U-Test**
	Techn. Affinity	No	Yes	T-Test*	No	No	U-test
	Job	X	X	X	No	Yes	U-Test**
Implement. Knowledge							
	Age	X	X	X	No	No	U-test
	Gender	X	X	X	No	Yes	U-Test**
	Program	X	X	X	No	Yes	U-Test**
	Techn. Affinity	X	X	X	No	Yes	U-Test**
	Job	X	X	X	No	Yes	U-Test**
Process Knowledge							
	Age	X	X	X	No	Yes	T-Test*
	Gender	X	X	X	Yes	Yes	T-test
	Program	X	X	X	Yes	No	Welch-Test
	Techn. Affinity	X	X	X	Yes	Yes	T-test
	Job	X	X	X	No	Yes	U-Test**
Technology Skills							
	Age	No	Yes	T-Test*	No	Yes	T-Test*
	Gender	No	Yes	T-Test*	Yes	Yes	T-test
	Program	No	Yes	T-Test*	Yes	Yes	T-test
	Techn. Affinity	No	No	U-Test	No	Yes	U-Test**
	Job	X	X	X	Yes	Yes	T-test

* T-test applicable, because sizes of group 1 and 2 > 30
** T-test not applicable, because sizes of group 1 and 2 <=30

Table S6: Results of Shapiro-Wilk- and Levene-Tests and corresponding selection of the statistical test regarding the relevance of competencies by groups (Part 2)

		**Patients**			**Therapists**		
Competency	Group Variable	Normal Distribution	Equal variances	Statistical test	Normal Distribution	Equal variances	Statistical test
Adaptability							
	Age	No	Yes	T-Test*	No	Yes	T-Test*
	Gender	No	Yes	T-Test*	No	Yes	U-Test**
	Program	No	Yes	T-Test*	No	Yes	U-Test**
	Techn. Affinity	No	Yes	T-Test*	No	Yes	U-Test**
	Job	X	X	X	No	Yes	U-Test**
Reflectivity							
	Age	No	No	U-Test	No	Yes	T-Test*
	Gender	No	Yes	T-Test*	No	Yes	U-Test**
	Program	No	Yes	T-Test*	No	Yes	U-Test**
	Techn. Affinity	No	Yes	T-Test*	No	Yes	U-Test**
	Job	X	X	X	No	Yes	U-Test**
Analytic Skills							
	Age	No	Yes	T-Test*	No	Yes	T-Test*
	Gender	No	Yes	T-Test*	No	Yes	U-Test**
	Program	No	Yes	T-Test*	No	Yes	U-Test**
	Techn. Affinity	No	Yes	T-Test*	No	Yes	U-Test**
	Job	X	X	X	No	Yes	U-Test**
Empathic Capacity							
	Age	No	Yes	T-Test*	No	No	U-test
	Gender	No	Yes	T-Test*	No	Yes	U-Test**
	Program	No	No	U-Test	No	Yes	U-Test**
	Techn. Affinity	No	Yes	T-Test*	No	Yes	U-Test**
	Job	X	X	X	No	No	U-test
Teamwork Skills							
	Age	No	No	U-Test	No	Yes	T-Test*
	Gender	No	Yes	T-Test*	Yes	Yes	T-test
	Program	No	No	U-Test	Yes	Yes	T-test
	Techn. Affinity	No	Yes	T-Test*	Yes	Yes	T-test
	Job	X	X	X	No	Yes	U-Test**
Communic. Skills							
	Age	No	Yes	T-Test*	No	No	U-test
	Gender	No	Yes	T-Test*	No	Yes	U-Test**
	Program	No	No	U-Test	No	Yes	U-Test**
	Techn. Affinity	No	Yes	T-Test*	No	Yes	U-Test**
	Job	X	X	X	No	Yes	U-Test**
Motivational Skills							
	Age	No	No	U-Test	No	Yes	T-Test*
	Gender	No	Yes	T-Test*	No	Yes	U-Test**
	Program	No	Yes	T-Test*	No	Yes	U-Test**
	Techn. Affinity	No	Yes	T-Test*	No	Yes	U-Test**
	Job	X	X	X	No	Yes	U-Test**
Self-Management							
	Age	No	No	U-Test	No	Yes	T-Test*
	Gender	No	Yes	T-Test*	No	Yes	U-Test**
	Program	No	No	U-Test	No	Yes	U-Test**
	Techn. Affinity	No	Yes	T-Test*	No	Yes	U-Test**
	Job	X	X	X	No	Yes	U-Test**

* T-test applicable, because sizes of group 1 and 2 > 30
** T-test not applicable, because sizes of group 1 and 2 <=30

Table S6: Results of Shapiro-Wilk- and Levene-Tests and corresponding selection of the statistical test regarding the relevance of competencies by groups (Part 3)

		**Patients**			**Therapists**		
Competency	Group Variable	Normal Distribution	Equal variances	Statistical test	Normal Distribution	Equal variances	Statistical test
Patience							
	Age	No	No	U-Test*	No	Yes	T-Test*
	Gender	No	Yes	T-Test*	No	Yes	U-Test**
	Program	No	Yes	T-Test*	No	Yes	U-Test**
	Techn. Affinity	No	No	U-Test*	No	Yes	U-Test**
	Job	X	X	X	No	Yes	U-Test**
Self-awareness							
	Age	No	No	U-Test*	No	Yes	T-Test*
	Gender	No	Yes	T-Test*	No	Yes	U-Test**
	Program	No	Yes	T-Test*	No	Yes	U-Test**
	Techn. Affinity	No	No	U-Test*	No	Yes	U-Test**
	Job	X	X	X	No	Yes	U-Test**
Reading/writing Skills							
	Age	No	Yes	T-Test*	X	X	X
	Gender	No	Yes	T-Test*	X	X	X
	Program	No	Yes	T-Test*	X	X	X
	Techn. Affinity	No	Yes	T-Test*	X	X	X
	Job	X	X	X	X	X	X
Therapeutic-professional Skills							
	Age	X	X	X	No	No	U-test
	Gender	X	X	X	No	Yes	U-Test**
	Program	X	X	X	No	Yes	U-Test**
	Techn. Affinity	X	X	X	No	No	U-test
	Job	X	X	X	No	No	U-test
Technology Affinity							
	Age	No	Yes	T-Test*	Yes	Yes	T-test
	Gender	No	Yes	T-Test*	Yes	Yes	T-test
	Program	No	Yes	T-Test*	Yes	Yes	T-test
	Techn. Affinity	No	Yes	T-Test*	No	Yes	U-Test**
	Job	X	X	X	Yes	Yes	T-test
Technology Acceptance							
	Age	No	Yes	T-Test*	No	Yes	T-Test*
	Gender	No	Yes	T-Test*	No	No	U-test
	Program	No	Yes	T-Test*	No	Yes	U-Test**
	Techn. Affinity	No	Yes	T-Test*	No	Yes	U-Test**
	Job	X	X	X	No	Yes	U-Test**
Willingness to learn							
	Age	No	Yes	T-Test*	No	No	U-test
	Gender	No	Yes	T-Test*	No	Yes	U-Test**
	Program	No	Yes	T-Test*	No	Yes	U-Test**
	Techn. Affinity	No	Yes	T-Test*	No	Yes	U-Test**
	Job	X	X	X	No	Yes	U-Test**

* T-test applicable, because sizes of group 1 and 2 > 30
** T-test not applicable, because sizes of group 1 and 2 <=30

Table S6: Results of Shapiro-Wilk- and Levene-Tests and corresponding selection of the statistical test regarding the relevance of competencies by groups (Part 4)

		**Patients**			**Therapists**		
Competency	Group Variable	Normal Distribution	Equal variances	Statistical test	Normal Distribution	Equal variances	Statistical test
Open-mindedness							
	Age	No	No	U-Test	No	No	U-test
	Gender	No	Yes	T-Test	No	Yes	U-Test**
	Program	No	Yes	T-Test	No	Yes	U-Test**
	Techn. Affinity	No	No	U-Test	No	Yes	U-Test**
	Job	X	X	X	No	Yes	U-Test**
Frustrat. tolerance							
	Age	No	Yes	T-Test*	No	Yes	T-Test*
	Gender	No	Yes	T-Test*	No	Yes	U-Test**
	Program	No	Yes	T-Test*	No	Yes	U-Test**
	Techn. Affinity	No	Yes	T-Test*	No	Yes	U-Test**
	Job	X	X	X	No	No	U-test
Self-efficacy expectation							
	Age	No	Yes	T-Test*	No	Yes	T-Test*
	Gender	No	Yes	T-Test*	No	Yes	U-Test**
	Program	No	Yes	T-Test*	No	Yes	U-Test**
	Techn. Affinity	No	Yes	T-Test*	No	Yes	U-Test**
	Job	X	X	X	No	Yes	U-Test**
Self-interest in the program							
	Age	No	Yes	T-Test*	No	No	U-test
	Gender	No	Yes	T-Test*	No	Yes	U-Test**
	Program	No	Yes	T-Test*	No	Yes	U-Test**
	Techn. Affinity	No	Yes	T-Test*	No	No	U-test
	Job	X	X	X	No	Yes	U-Test**
Experience in analoge therapy							
	Age	No	Yes	T-Test*	No	No	U-test
	Gender	No	Yes	T-Test*	No	No	U-test
	Program	No	Yes	T-Test*	No	Yes	U-Test**
	Techn. Affinity	No	Yes	T-Test*	No	No	U-test
	Job	X	X	X	No	No	U-test
Experience with digital health apps / digital work apps							
	Age	No	Yes	T-Test*	No	Yes	T-Test*
	Gender	No	Yes	T-Test*	Yes	Yes	T-test
	Program	No	Yes	T-Test*	Yes	Yes	T-test
	Techn. Affinity	No	Yes	T-Test*	Yes	Yes	T-test
	Job	X	X	X	Yes	Yes	T-test
Experience with digital tools							
	Age	No	Yes	T-Test*	No	Yes	T-Test*
	Gender	No	Yes	T-Test*	No	Yes	U-Test**
	Program	No	Yes	T-Test*	No	Yes	U-Test**
	Techn. Affinity	No	Yes	T-Test*	No	Yes	U-Test**
	Job	X	X	X	No	No	U-test

* T-test applicable, because sizes of group 1 and 2 > 30
** T-test not applicable, because sizes of group 1 and 2 <=30
