# Supplementary material for: Developing a competency model for telerehabilitation therapists and patients: Results of a cross-sectional online survey
Source: PLOS Digit Health. 2025 Jan 3;4(1):e0000710. doi: 10.1371/journal.pdig.0000710 (PMC11698311; doi:10.1371/journal.pdig.0000710)
Supplement: S7 Appendix — (PDF) [file pdig.0000710.s007.pdf]

S7 appendix: Telerehabilitation steps

Table S7.1: Steps undertaken by patients

		**Patients (all, n=262)**		**Patients (video user, n=113)**		**Patients (app user, n=149)**	
	Variable	n	%	n	%	n	%
Steps for preparation							
	Independent information about the program	138	52.7	67	59.3	71	47.7
	Participation in a personal conversation or presentation with information about the program	128	48.9	51	45.1	77	51.7
	Participation in a practical introduction to the program	72	27.5	11	9.7	61	40.9
	Technical setup of the program	50	19.1	22	19.5	28	18.8
	Individual adaptation of the therapy to own needs (if necessary with therapist)	91	34.7	25	22.1	66	44.3
Steps during therapy							
	Following the therapist's instructions	184	70.2	76	67.3	108	72.5
	Adapting the therapy to individual needs (e.g. selecting content, difficulty)	135	51.5	51	45.1	84	56.4
	Dealing with health problems (e.g. certain symptoms or emergencies)	103	39.3	62	54.9	41	27.5
	Solving technical problems	42	16.0	24	21.2	18	12.1
	Self-monitoring (e.g. therapy progress, health parameters)	82	31.3	35	31.0	47	31.5
	Motivating oneself	165	63.0	75	66.4	90	60.4
	Reminding oneself (e.g. of therapy execution or appointments)	131	50.0	57	50.4	74	49.6
	Own documentation of therapy sessions	44	16.8	25	22.1	49.6	12.8
Responsible							
	Responsibility for some steps lies with others	90	34.4	45	39.8	45	30.2

Table S7.2: Steps undertaken by therapists

		Therap. (all, n=73)		Therap. (video user, n=15)		Therap. (app user, n=58		Therap. (tele, n=15)		Therap. (on-side, n=58)	
	Variable	n	%	n	%	n	%	n	%	n	%
Steps for preparation											
	Informing patients about the program	66	90.4	12	80.0	54	93.1	14	93.3	52	89.7
	Practical instruction of patients in the program	54	74.0	7	46.7	47	81.0	8	53.3	46	79.3
	Technical setup of the program	27	37.0	2	13.3	25	43.1	6	40.0	21	36.2
	Supporting patients with the technical setup	53	72.6	6	40.0	47	81.0	11	73.3	42	72.4
	Individual adaptation of the therapy to the patient's needs	63	86.3	11	73.3	52	89.7	12	80.0	51	87.9
Steps during therapy	Variable										
	Guiding patients	55	75.3	9	60.0	46	79.3	13	86.7	42	72.4
	Adapting the therapy to the patient's needs	63	86.3	14	93.3	49	84.5	13	86.7	50	86.2
	Supporting patients with health problems	48	65.8	11	73.3	37	63.8	11	73.3	37	63.8
	Solving technical problems	38	52.1	5	33.3	33	56.9	10	66.7	28	48.3
	Monitoring patients (e.g. therapy progress, health parameters)	48	65.8	7	46.7	41	70.7	13	86.7	35	60.3
	Providing feedback or motivation	54	74.0	11	73.3	43	74.1	15	100.0	39	67.2
	Reminding patients (e.g. of therapy execution or appointments)	51	69.9	8	53.3	43	74.1	13	86.7	38	65.5
	Documentation of therapy sessions	41	56.2	12	80.0	29	50.0	12	80.0	29	50.0
Responsible											
	Responsibility for some steps lies with others	18	24.7	6	40.0	12	20.7	0	0.0	18	31.0

* Additional analysis:
54.2% of male therapists carry out a “Technical setup of the program”
