# Supplementary material for: Developing a competency model for telerehabilitation therapists and patients: Results of a cross-sectional online survey
Source: PLOS Digit Health. 2025 Jan 3;4(1):e0000710. doi: 10.1371/journal.pdig.0000710 (PMC11698311; doi:10.1371/journal.pdig.0000710)
Supplement: S8 Appendix — (PDF) [file pdig.0000710.s008.pdf]

S8 appendix: Relevance of competencies

Table S8.1: Relevance of competencies accessed by patients

	**Patients (all, n=262)**		**Patients (video user, n=113)**		**Patients (app user, n=149)**	
Competency	M	SD	M	SD	M	SD
Telerehab. Knowlege	5.3	.0973	5.1	.1543	5.6	.1225
Legal Knowledge	5.0	.1155	5.1	.1740	4.9	.1546
Technology Knowledge	5.2	.1026	5.1	.1594	5.2	.1342
Medical Knowledge	5.4	.1052	5.2	.1720	5.6	.1289
Implement. Knowledge	X	X	X	X	X	X
Process Knowledge	X	X	X	X	X	X
**Knowledge Index**	5.2	.0771	5.1	.1221	5.2	.0986
Technology Skills	5.2	.0936	5.2	.1380	5.3	.1271
Adaptability	5.4	.0835	5.3	.1249	5.5	.1117
Reflectivity	5.7	.0849	5.8	.1235	5.7	.1165
Analytic Skills	5.4	.0953	5.4	.1294	5.3	.1360
Empathic Capacity	5.4	.1090	5.7	.1287	5.1	.1614
Teamwork Skills	5.3	.1128	5.7	.1315	5.0	.16780
Communic. Skills	5.7	.0994	6.0	.1166	5.5	.1486
Motivational Skills	5.7	.0851	5.5	.1306	5.9	.1108
Self-Management	5.8	.0823	5.4	.1380	6.2	.0909
Patience	5.5	.0966	5.4	.1431	5.6	.1306
Self-awareness	5.9	.0797	5.9	.1211	5.9	.1061
Reading/writing Skills	5.1	.1140	4.6	.1771	5.5	.1411
Therapeutic-professional Skills	X	X	X	X	X	X
**Skills Index**	5.5	.0674	5.5	.0975	5.5	.0929
Technology Affinity	4.7	.0929	4.4	.1274	4.9	.1288
Technology Acceptance	5.3	.0871	5.2	.1361	5.4	.1128
Willingness to learn	5.7	.0806	5.5	.1238	6.0	.1030
Open-mindedness	5.8	.0773	5.7	.1129	5.9	.1050
Frustrat. tolerance	5.2	.0927	5.0	.1339	5.3	.1271
Self-efficacy expectation	5.3	.0842	5.1	.1248	5.5	.1125
Self-interest in the program	6.3	.0661	6.2	.1005	6.4	.0875
**Attitude Index**	5.5	.0628	5.3	.0918	5.6	.0840
Experience in analoge therapy	3.9	.1262	3.7	.1913	4.1	.1666
Experience with digital health apps	3.5	.1177	3.1	.1699	3.9	.1569
Experience with digital tools	5.0	.0994	4.9	.1394	5.2	.1389
**Experience Index**	4.2	.0921	3.9	.1353	4.4	.1226

Table S8.2: Relevance of competencies accessed by therapists

	**Therapists (all, n=73)**		**Therapists (video user, n=15)**		**Therapists (app user, n=58)**		**Therapists (tele, n=15)**		**Therapists (on-side, n=58)**	
Competency	M	SD	M	SD	M	SD	M	SD	M	SD
Telerehab. Knowledge	6.1	.1870	5.7	.4415	6.1	.2064	6.3	.3473	6.0	.2179
Legal Knowledge	5.1	.1896	5.2	.4493	5.1	.2106	5.5	.3763	5.0	.2179
Technology Knowledge	4.7	.1928	4.5	.3887	4.8	.2218	5.3	.3157	4.6	.2257
Medical Knowledge	6.1	.1650	6.4	.2895	6.0	.1936	6.4	.2350	6.0	.1982
Implement. Knowledge	5.4	.1915	5.1	.3065	5.4	.2281	5.5	.3763	5.3	.2233
Process Knowledge	4.4	.1897	4.5	.2153	4.3	.2049	3.9	.3838	4.5	.1861
**Knowledge Index**	**5.3**	**.1328**	**5.2**	**.2535**	**5.3**	**.1548**	**5.5**	**.1803**	**5.2**	**.1606**
Technology Skills	4.6	.2160	4.9	.4458	4.6	.2474	4.6	.4557	4.6	.2470
Adaptability	5.7	.1780	6.1	.3960	5.6	.1993	5.7	.3157	5.7	.2098
Reflectivity	5.5	.1800	5.5	.4239	5.5	.2002	5.9	.2364	5.4	.2171
Analytic Skills	5.5	.1768	5.7	.4328	5.4	.1939	5.4	.3055	5.5	.2090
Empathic Capacity	5.9	.1894	6.0	.4781	5.8	.2060	6.8	.1447	5.6	.2252
Teamwork Skills	4.6	.2262	4.5	.4563	4.7	.2607	5.3	.5561	4.5	.2438
Communic. Skills	5.8	.1918	6.1	.4079	5.8	.2183	6.7	.1869	5.6	.2290
Motivational Skills	5.8	.1904	5.6	.3754	5.8	.2202	6.4	.2545	5.6	.2265
Self-Management	5.8	.1874	5.5	.4768	5.8	.2025	5.9	.3581	5.7	.2180
Patience	5.3	.1943	5.6	.4231	5.3	.2197	5.4	.3055	5.3	.2325
Self-awareness	5.2	.2071	5.5	.5243	5.1	.2239	5.2	.3581	5.1	.2448
Reading/writing Skills	X	X	X	X	X	X	X	X	X	X
Therapeutic-professional Skills	6.3	.1725	6.3	.4102	6.3	.1914	6.7	.1260	6.3	.2138
**Skills Index**	**5.5**	**.1545**	**5.6**	**.3683**	**5.5**	**.1711**	**5.8**	**.1706**	**5.4**	**.1884**
Technology Affinity	4.5	.1913	4.1	.3581	4.6	.2211	4.3	.3712	4.6	.2215
Technology Acceptance	5.6	.1750	5.3	.3333	5.7	.2028	6.3	.2840	5.5	.2031
Willingness to learn	5.7	.1723	5.3	.3333	5.8	.1980	6.3	.2667	5.6	.2027
Open-mindedness	6.0	.1711	5.8	.3677	6.0	.1944	6.2	.2960	5.9	.2018
Frustrat. tolerance	5.1	.1707	5.6	.4000	5.0	.1865	5.1	.2282	5.1	.2074
Self-efficacy expectation	5.4	.1723	5.5	.3501	5.4	.1985	5.5	.3066	5.4	.2029
Self-interest in the program	5.2	.2027	5.1	.4239	5.2	.2321	5.7	.3187	5.1	.2401
**Attitude Index**	**5.4**	**.1458**	**5.2**	**.2919**	**5.4**	**.1682**	**5.6**	**.2098**	**5.3**	**.1751**
Experience in analoge therapy	5.6	.2117	5.3	.5040	5.7	.2338	5.9	.3838	5.5	.2474
Experience with digital work apps	4.3	.1961	4.9	.4768	4.1	.2111	4.1	.3581	4.3	.2297
Experience with digital tools	5.2	.1844	5.3	.3712	5.2	.2127	5.4	.1902	5.1	.2269
**Experience Index**	**5.0**	**.1673**	**5.2**	**.4177**	**5.0**	**.1824**	**5.1**	**.1863**	**5.0**	**.2054**
