# Supplementary material for: Developing a competency model for telerehabilitation therapists and patients: Results of a cross-sectional online survey
Source: PLOS Digit Health. 2025 Jan 3;4(1):e0000710. doi: 10.1371/journal.pdig.0000710 (PMC11698311; doi:10.1371/journal.pdig.0000710)
Supplement: S9 Appendix — (PDF) [file pdig.0000710.s009.pdf]

S7 appendix: Results of T-Tests and U-Tests

Table S7: Results of T-Tests and U-Tests regarding the relevance of competencies by groups (Part 1)

		Patients		Therapists	
Competency	Group Variable	Statistical Test	p-value	Statistical Test	p-value
Telerehab. Knowlege					
	Age	T-Test	.0913	U-Test	.0094*
	Gender	T-Test	.6923	U-Test	.2116
	Program	T-Test	.0143*	U-Test	.1665
	Techn. Affinity	T-Test	.6212	U-Test	.9046
	Job	X	X	U-Test	.3836
Legal Knowledge					
	Age	T-Test	.2048	T-Test	.2602
	Gender	T-Test	.8768	U-Test	.4014
	Program	T-Test	.5056	U-Test	.6912
	Techn. Affinity	T-Test	.0402*	Welch-Test	.0418*
	Job	X	X	U-Test	.3761
Technology Knowledge					
	Age	T-Test	.9852	U-Test	.0171*
	Gender	T-Test	.5445	T-Test	.2154
	Program	T-Test	.7015	T-Test	.5444
	Techn. Affinity	T-Test	.3856	U-Test	.0630
	Job	X	X	T-Test	.1351
Medical Knowledge					
	Age	T-Test	.1920	U-Test	.9219
	Gender	T-Test	.6392	U-Test	.7248
	Program	U-Test	.0357*	U-Test	.3170
	Techn. Affinity	T-Test	.7252	U-Test	.0231*
	Job	X	X	U-Test	.5492
Implement. Knowledge					
	Age	X	X	U-Test	.2017
	Gender	X	X	U-Test	.8469
	Program	X	X	U-Test	.1967
	Techn. Affinity	X	X	U-Test	.8167
	Job	X	X	U-Test	.9776
Process Knowledge					
	Age	X	X	T-Test	.3365
	Gender	X	X	T-Test	.7158
	Program	X	X	Welch-Test	.4923
	Techn. Affinity	X	X	T-Test	.5898
	Job	X	X	U-Test	.2331
Technology Skills					
	Age	T-Test	.7618	T-Test	.0667
	Gender	T-Test	.9569	T-Test	.4861
	Program	T-Test	.4948	T-Test	.5595
	Techn. Affinity	U-Test	.1677	U-Test	.5902
	Job	X	X	T-Test	.9695

* significant result

Table S7: Results of T-Tests and U-Tests regarding the relevance of competencies by groups (Part 2)

		Patients		Therapists	
Competency	Group Variable	Statistical Test	p-value	Statistical Test	p-value
Adaptability					
	Age	T-Test	.0088*	T-Test	.3930
	Gender	T-Test	.4435	U-Test	.9951
	Program	T-Test	.1667	U-Test	.1778
	Techn. Affinity	T-Test	.6629	U-Test	.9434
	Job	X	X	U-Test	.6310
Reflectivity					
	Age	U-Test	.5504	T-Test	.5724
	Gender	T-Test	.4955	U-Test	.6792
	Program	T-Test	.6095	U-Test	.8431
	Techn. Affinity	T-Test	.0639	U-Test	.2801
	Job	X	X	U-Test	.5525
Analytic Skills					
	Age	T-Test	.0752	T-Test	.8466
	Gender	T-Test	.9462	U-Test	.1937
	Program	T-Test	.4880	U-Test	.3691
	Techn. Affinity	T-Test	.2537	U-Test	.2036
	Job	X	X	U-Test	.3431
Empathic Capacity					
	Age	T-Test	.0642	U-Test	.2802
	Gender	T-Test	.7725	U-Test	.8045
	Program	U-Test	.0223*	U-Test	.2983
	Techn. Affinity	T-Test	.2138	U-Test	.5919
	Job	X	X	U-Test	.0026*
Teamwork Skills					
	Age	U-Test	.0152*	T-Test	.0391*
	Gender	T-Test	.5397	T-Test	.2423
	Program	U-Test	.0321*	T-Test	.7160
	Techn. Affinity	T-Test	.0915	T-Test	.7806
	Job	X	X	U-Test	.0723
Communic. Skills					
	Age	T-Test	.3424	U-Test	.2011
	Gender	T-Test	.9819	U-Test	.8651
	Program	U-Test	.1256	U-Test	.5436
	Techn. Affinity	T-Test	.2176	U-Test	.3584
	Job	X	X	U-Test	.0100*
Motivational Skills					
	Age	U-Test	.6297	T-Test	.5049
	Gender	T-Test	.9680	U-Test	.4339
	Program	T-Test	.0328*	U-Test	.1769
	Techn. Affinity	T-Test	.0970	U-Test	.7839
	Job	X	X	U-Test	.0625
Self-Management					
	Age	U-Test	.9221	T-Test	.5946
	Gender	T-Test	.1252	U-Test	.1779
	Program	U-Test	0.0000*	U-Test	.5784
	Techn. Affinity	T-Test	.3883	U-Test	.3907
	Job	X	X	U-Test	.6967

* significant result

Table S7: Results of T-Tests and U-Tests regarding the relevance of competencies by groups (Part 3)

		**Patients**		**Therapists**	
Competency	Group Variable	Statistical Test	p-value	Statistical Test	p-value
Patience					
	Age	U-Test	.2302	T-Test	.7814
	Gender	T-Test	.3072	U-Test	.7580
	Program	T-Test	.3597	U-Test	.4823
	Techn. Affinity	U-Test	.0061*	U-Test	.5502
	Job	X	X	U-Test	.6179
Self-awareness					
	Age	U-Test	.7848	T-Test	.2762
	Gender	T-Test	.8564	U-Test	.8326
	Program	T-Test	.5861	U-Test	.1800
	Techn. Affinity	U-Test	.9828	U-Test	.1021
	Job	X	X	U-Test	.8223
Reading/writing Skills					
	Age	T-Test	.2503	X	X
	Gender	T-Test	.6719	X	X
	Program	T-Test	.0001*	X	X
	Techn. Affinity	T-Test	.6741	X	X
	Job	X	X	X	X
Therapeutic-professional Skills					
	Age	X	X	U-Test	.6985
	Gender	X	X	U-Test	.6424
	Program	X	X	U-Test	.9445
	Techn. Affinity	X	X	U-Test	.2872
	Job	X	X	U-Test	.8961
Technology Affinity					
	Age	T-Test	.2503	T-Test	.4546
	Gender	T-Test	.4259	T-Test	.8605
	Program	T-Test	.0038*	T-Test	.2445
	Techn. Affinity	T-Test	.0695	U-Test	.6577
	Job	X	X	T-Test	.5268
Technology Acceptance					
	Age	T-Test	.5388	T-Test	.0279*
	Gender	T-Test	.0625	U-Test	.4475
	Program	T-Test	.1739	U-Test	.1125
	Techn. Affinity	T-Test	.4941	U-Test	.9150
	Job	X	X	U-Test	.0216*
Willingness to learn					
	Age	T-Test	.1314	U-Test	.0576
	Gender	T-Test	.7510	U-Test	.8347
	Program	T-Test	.0020*	U-Test	.0656
	Techn. Affinity	T-Test	.1553	U-Test	.8228
	Job	X	X	U-Test	.1008

* significant result

Table S7: Results of T-Tests and U-Tests regarding the relevance of competencies by groups (Part 4)

		**Patients**		**Therapists**	
Competency	Group Variable	Statistical Test	p-value	Statistical Test	p-value
Open-mindedness					
	Age	U-Test	.7998	U-Test	.2648
	Gender	T-Test	.7136	U-Test	.9346
	Program	T-Test	.1013	U-Test	.4156
	Techn. Affinity	U-Test	.0239*	U-Test	.5774
	Job	X	X	U-Test	.5673
Frustrat. tolerance					
	Age	T-Test	.2639	T-Test	.4105
	Gender	T-Test	.6050	U-Test	.9904
	Program	T-Test	.1729	U-Test	.1080
	Techn. Affinity	T-Test	.0679	U-Test	.2708
	Job	X	X	U-Test	.4960
Self-efficacy expectation					
	Age	T-Test	.3174	T-Test	.4623
	Gender	T-Test	.1340	U-Test	.5886
	Program	T-Test	.0320*	U-Test	.9146
	Techn. Affinity	T-Test	.5161	U-Test	.1073
	Job	X	X	U-Test	.9374
Self-interest in the program					
	Age	T-Test	.8378	U-Test	.4117
	Gender	T-Test	.4789	U-Test	.9233
	Program	T-Test	.1915	U-Test	.6796
	Techn. Affinity	T-Test	.8256	U-Test	.0211*
	Job	X	X	U-Test	.3931
Experience in analoge therapy					
	Age	T-Test	.3957	U-Test	.2189
	Gender	T-Test	.0066*	U-Test	.1022
	Program	T-Test	.0611	U-Test	.3439
	Techn. Affinity	T-Test	.1214	U-Test	.0893
	Job	X	X	U-Test	.7039
Experience with digital health apps / digital work apps					
	Age	T-Test	.4468	T-Test	.1286
	Gender	T-Test	.3270	T-Test	.9711
	Program	T-Test	.0009*	T-Test	.1164
	Techn. Affinity	T-Test	.0093*	T-Test	.4402
	Job	X	X	T-Test	.6189
Experience with digital tools					
	Age	T-Test	.9858	T-Test	.4822
	Gender	T-Test	.5788	U-Test	.9952
	Program	T-Test	.2104	U-Test	1.0000
	Techn. Affinity	T-Test	.3613	U-Test	.0423
	Job	X	X	U-Test	.9944

* significant result
